# Linear antenna array optimization using flower pollination algorithm

**DOI:** 10.1186/s40064-016-1961-7

**Published:** 2016-03-10

**Authors:** Prerna Saxena, Ashwin Kothari

**Affiliations:** Department of Electronics and Communication Engineering, Visvesvaraya National Institute of Technology, South Ambazari Road, Nagpur, Maharashtra 440010 India

**Keywords:** Linear antenna array, Flower pollination algorithm, Evolutionary algorithms, Antenna array optimization, Side lobe level, Null placement

## Abstract

Flower pollination algorithm (FPA) is a new nature-inspired evolutionary algorithm used to solve multi-objective optimization problems. The aim of this paper is to introduce FPA to the electromagnetics and antenna community for the optimization of linear antenna arrays. FPA is applied for the first time to linear array so as to obtain optimized antenna positions in order to achieve an array pattern with minimum side lobe level along with placement of deep nulls in desired directions. Various design examples are presented that illustrate the use of FPA for linear antenna array optimization, and subsequently the results are validated by benchmarking along with results obtained using other state-of-the-art, nature-inspired evolutionary algorithms such as particle swarm optimization, ant colony optimization and cat swarm optimization. The results suggest that in most cases, FPA outperforms the other evolutionary algorithms and at times it yields a similar performance.

## Background

Antenna arrays are widely used in wireless communications due to their ability to enhance performance by providing high gain, high directivity, increased spectrum efficiency and beam steering capability (Balanis 1997). Due to increased electromagnetic pollution, null placement in the field of interferences has gained much importance (Vescovo 2000). Specifically, null placement is of critical importance to radar, sonar and wireless communication systems as it minimizes the degradation of signal-to-noise ratio performance due to undesired interference (Akdagli 2001). Thus, null placement along with suppression of side lobe level (SLL) is key to the design of antenna arrays.

Extensive study of linear antenna array synthesis has been reported in the literature (Er 1990; Panduro et al. 2005; Bianchi et al. 2014). For optimal pattern synthesis of linear array, SLL minimization and null placement can be achieved in two ways: either by optimizing the excitation amplitude and phase while at the same time maintaining uniform spacing similar to conventional array or by optimizing element spacing and assuming uniform amplitude and phase excitation. Various evolutionary algorithms such as genetic algorithm (GA) (Zhang et al. 2014; Goswami and Mandal 2013), simulated annealing (SA) (Murino et al. 1996), particle swarm optimization (PSO) (Khodier and Christodoulou 2005; Li et al. 2010; Jin and Rahmat-Samii 2007), ant colony optimization (ACO) (Rajo-Iglesias and Quevedo-Teruel 2007), invasive weed optimization (IWO) (Karimkashi and Kishk 2010) and cat swarm optimization (CSO) (Pappula and Ghosh 2014) have been successfully applied for the optimization of linear arrays.

In this paper, a new nature-inspired evolutionary algorithm, flower pollination algorithm (FPA) (Yang 2012; Yang et al. 2014) is proposed for linear antenna array optimization. FPA is a metaheuristic algorithm inspired by the pollination process of flowering plants. It was developed by Xin-She Yang in 2012 (Yang 2012). FPA has been applied to solve practical optimization problems in engineering (Yang et al. 2014) such as disc brake design, spring design optimization, welded beam design, speed reducer design and pressure vessel design. FPA has also been used in areas like solar PV parameter estimation (Alam et al. 2015), fuzzy selection for dynamic economic dispatch (Dubey et al. 2015), etc. However, to the best of the authors’ knowledge, this is the first time that FPA is being proposed for linear antenna array synthesis. In this paper, FPA is applied to linear antenna array in order to obtain array pattern with minimum SLL. In addition, nulls are placed in desired directions by optimizing the spacing between the antenna array elements. Furthermore, in this paper, the design problem of minimization of peak SLL, and that of imposing deeper nulls in the interference directions under the constraints of a reduced SLL of linear antenna arrays is modeled as an optimization problem. To solve this design goal, the flower pollination algorithm (FPA) is used to determine optimum antenna positions in the array.

This section has presented a brief introduction to linear antenna array, the FPA and its applications in optimization problems, and the main objective of this work. The rest of the paper is organized as follows: the linear antenna array geometry, configuration and array factor equations are discussed in “Linear antenna array” section. “Flower pollination algorithm” section presents an elaborate description of the flower pollination algorithm along with a flowchart outlining the steps of FPA implementation. Various design examples for linear array synthesis, and the FPA optimized antenna locations and corresponding array patterns are put forward in “Results and discussion” section. The validation of the obtained results, when compared to other nature-inspired evolutionary algorithms, is also presented in this section. “Conclusion” section offers the conclusion.

## Linear antenna array

**Fig. 1:**
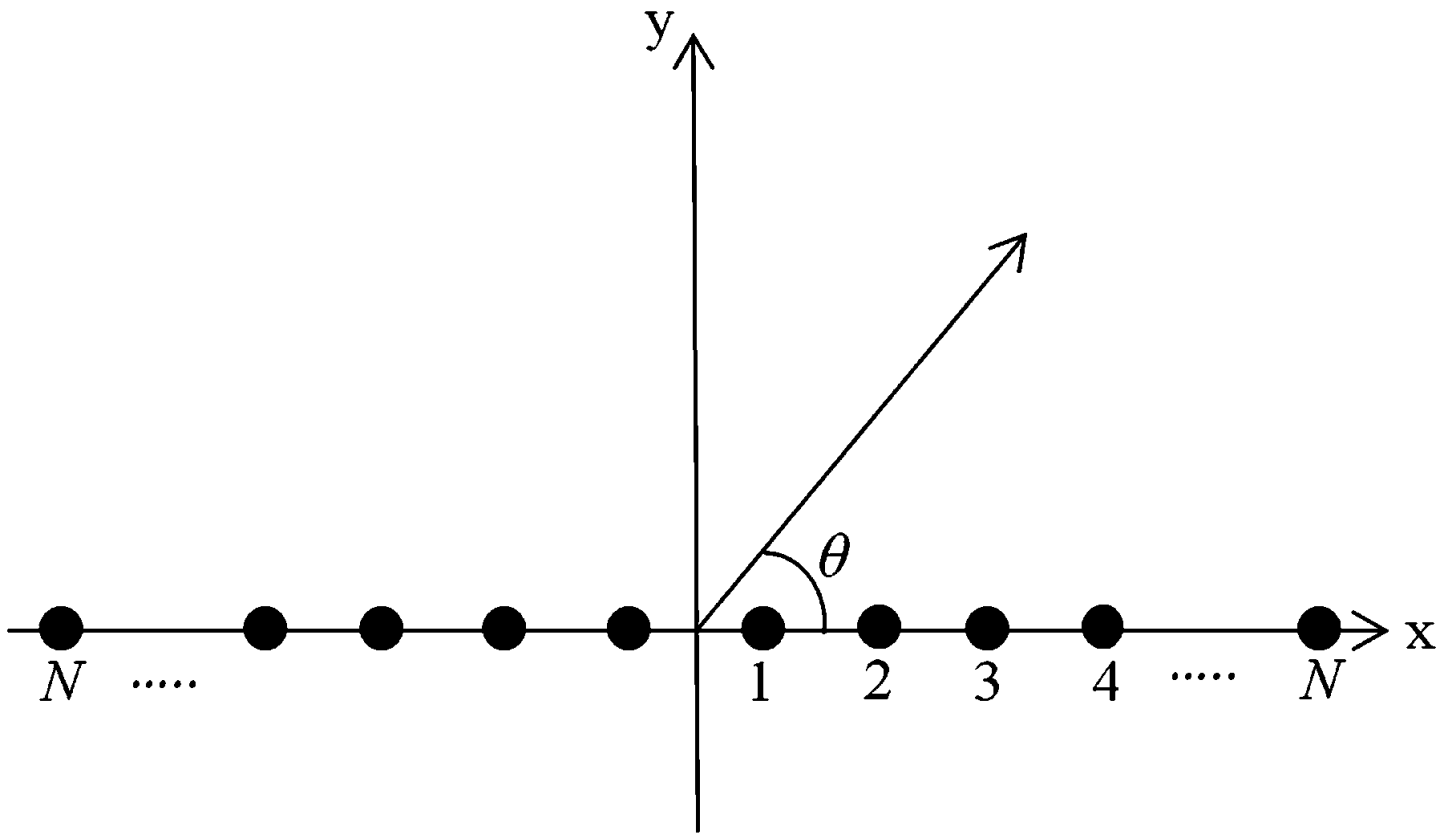
Antenna array geometry

A linear antenna array of *2N* isotropic elements placed symmetrically along the x-axis is considered in this work, as illustrated in Fig. 1.

Due to symmetry, the array factor (AF) (Balanis 1997; Khodier and Christodoulou 2005) in the azimuth plane is given by (1).

1 $$AF\left( \theta \right) = 2\sum\limits_{n = 1}^{N} {I_{n} } \cos {\kern 1pt} {\kern 1pt} \left( {kx_{n} \cos \left( \theta \right) + \psi_{n} } \right)$$

where $I_{n}$, $\psi_{n}$ and $x_{n}$ are the excitation amplitude, phase and position of *n*th element in the array. $k$ is the wave number and is given by 2π/λ and $\theta$ is the azimuth angle. It is assumed that the antenna array is subjected to uniform amplitude and phase excitation, that is, *I*_*n*_ = 1 and $\psi_{n}$ = 0. Thus, the AF in (1) gets modified to (2).

2 $$AF\left( \theta \right) = 2\sum\limits_{n = 1}^{N} {\cos {\kern 1pt} {\kern 1pt} \left( {kx_{n} \cos \left( \theta \right)} \right)}$$

The objective of this work is to apply the flower pollination algorithm to determine the optimized element positions,$x_{n}$, in order to achieve an array pattern with minimum SLL as well as placement of nulls in desired directions.

In linear antenna arrays, proper placement of antennas is very essential. If the antennas are placed too close to each other, it leads to mutual coupling effects. On the other hand, if the antennas are placed too far away, it leads to grating lobes. Thus, while solving this optimization problem, the following conditions must be satisfied:

(i) $$\left| {x_{i} - x_{j} } \right| > 0.25\lambda$$

(ii) $\hbox{min} \left\{ {x_{i} } \right\} > 0.125\lambda ;\,i = 1,2, \ldots ,N.\, i \ne j$ where, $x_{j}$ is the antenna position adjacent to the antenna position $x_{i}$ and $\left\{ {x_{i} } \right\}$ is the set of all antenna positions.

## Flower pollination algorithm

Inspired by the pollination process of flowering plants, the flower pollination algorithm (FPA) was developed by Xin-She Yang in 2012 (Yang 2012). FPA is extensively used for optimization of multi-objective real-world design problems (Yang et al. 2014). FPA is based on the following four rules (Yang 2012):

(i) Biotic and cross-pollination can be considered processes of global pollination, and pollen-carrying pollinators move in a way that obeys Levy flights.
(ii) For local pollination, abiotic pollination and self-pollination are used.
(iii) Pollinators, such as insects, can develop flower constancy. This in turn is equivalent to a reproduction probability that is proportional to the similarity of two flowers involved.
(iv) The interaction or switching of local pollination and global pollination can be controlled by a switch probability *p* ∈ [0, 1].

The basic parameters of FPA are defined as follows (Yang 2012; Yang et al. 2014):

1. Population Size (*n*): FPA is a population-based metaheuristic algorithm in which candidate solutions to the optimization problem play the role of individuals in a population, and the fitness function determines the quality of the solutions. Thus, FPA uses a population of (*n*) flowers/pollen gametes with random solutions as the starting point.
2. Switching Probability (*p*): Flower pollination activities can occur at both scales, local as well as global. However, the probability of local pollination is slightly higher than global pollination because adjacent flower patches or flowers in close vicinity are more likely to be pollinated by local flower pollen than those far away. To mimic this feature, a switching probability or proximity probability (*p*) can be effectively used to switch between common global pollination and intensive local pollination. This switching probability is slightly biased towards local pollination. During the execution of FPA, a random number between 0 and 1 is generated and is compared with the switching probability. If this number is less than (*p*), then global pollination is performed, otherwise local pollination is carried out.
3. *L(β)*: In the case of global pollination, flower pollen gametes are carried by pollinators, such as insects over long distances due to their ability to fly. The strength of the pollination is modelled by L(*β*) which is a step-size parameter, more specifically the Levy-flights-based step size. Since insects can travel extensively with various distance steps, a Levy flight is used to mimic this characteristic efficiently.
4. *γ*: It is used as a scaling factor to control the step size of the Levy flights for global pollination.
5. *ε*: For local pollination, pollen is selected from different flowers of the same plant species or from the same population. This mimics the flower constancy in a limited neighborhood. *ε* is drawn from a uniform distribution [0,1] so as to mimic a local random walk.

The parameters used in FPA along with their corresponding value/range are described in Table 1 (Yang et al. 2014).

**Table 1:** Parameters of FPA

Parameter	Description	Value/range
*n*	Population size	5–50
*p*	Switching probability	0.05–0.95
$\gamma$	Scaling factor (for step size)	0.1
*ε*	Uniform distribution	0–1
*L(β)*	Levy-flights based step size	>0, drawn from Levy distribution, 1 ≤ *β* ≤ 1.9

The implementation of FPA begins with the definition of the objective function and initialization of the population of flowers (*n*) with random solutions. The best solution in the initial population is computed. A switching probability [*p* ∈ (0, 1)] is defined. It controls the selection of either local pollination or global pollination. The choice between global pollination and local pollination is determined by generating a random number. If this random number is less than the switching probability (*p*), then global pollination is performed using (3). Otherwise, local pollination is carried out using (7).

The mathematical representation of global pollination [rule (i)] and flower constancy [rule (iii)] (Yang 2012) is given by (3).

3 $$x_{i}^{t + 1} = x_{i}^{t} + \gamma L\left( {g_{best} - x_{i}^{t} } \right)$$

where $x_{i}^{t}$ is the solution vector $x_{i}^{{}}$ at iteration *t*, and $g_{best}$ is the current best solution. $\gamma$ is a scaling factor to control step size. *L* denotes the Levy flights-based step size, which corresponds to the strength of the pollination. Since insects may travel over long distances with varying distance steps, a Levy flight can be used to model this characteristic efficiently. *L* is drawn from a Levy distribution by using (4).

4 $$L\sim\frac{{\beta \varGamma \left( \beta \right)\sin \left( {\pi \beta /2} \right)}}{\pi }\frac{1}{{s^{1 + \beta } }}$$

$\varGamma \left( \beta \right)$ is the standard gamma function. Mantegna proposed a fast and accurate algorithm to generate a stochastic variable whose probability density is close to a Levy stable distribution (Mantegna 1994). The required Levy stable stochastic process is generated in a single step by this algorithm. The pseudo-random step size (*s*) which obeys Levy distribution is drawn by using Mantegna algorithm for two Gaussian distributions *U* and *V* as follows in (5).

5 $$s = \frac{U}{{\left| V \right|^{{{1 \mathord{\left/ {\vphantom {1 \beta }} \right. \kern-0pt} \beta }}} }}$$

*U* and *V* are drawn from a Gaussian normal distribution with a zero mean and variance $\sigma^{2}$ given by (6).

6 $$\sigma^{2} = \left[ {\frac{{\varGamma \left( {1 + \beta } \right)}}{{\beta \varGamma \left( {{{\left( {1 + \beta } \right)} \mathord{\left/ {\vphantom {{\left( {1 + \beta } \right)} 2}} \right. \kern-0pt} 2}} \right)}} \cdot \frac{{\sin \left( {{{\pi \beta } \mathord{\left/ {\vphantom {{\pi \beta } 2}} \right. \kern-0pt} 2}} \right)}}{{2^{{{{\left( {\beta - 1} \right)} \mathord{\left/ {\vphantom {{\left( {\beta - 1} \right)} 2}} \right. \kern-0pt} 2}}} }}} \right]^{{{1 \mathord{\left/ {\vphantom {1 \beta }} \right. \kern-0pt} \beta }}}$$

For local pollination, the following mathematical formulation is used (Yang 2012).

7 $$x_{i}^{t + 1} = x_{i}^{t} + \varepsilon \left( {x_{j}^{t} - x_{k}^{t} } \right)$$

where $x_{j}^{t}$ and $x_{k}^{t}$ are pollen from different flowers of the same plant species. If $x_{j}^{t}$ and $x_{k}^{t}$ are selected from the same population, this is equivalent to a local random walk given that $\varepsilon$ is obtained from a uniform distribution in [0,1].

**Fig. 2:**
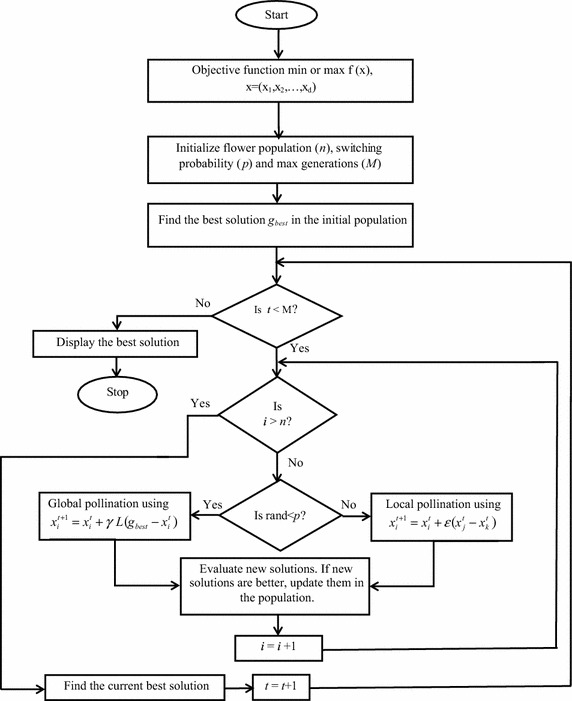
Flowchart of FPA

The basic steps of FPA are illustrated in the flowchart depicted in Fig. 2.

## Results and discussion

In this section, the FPA is applied to linear antenna array in order to determine the optimized antenna element positions to minimize the peak SLL, and to place nulls in desired directions. In design example A, the optimized antenna element locations are determined to minimize the peak SLL in the specified spatial region. Design examples B and C illustrate the application of FPA to determine the optimized antenna element positions in order to minimize SLL as well as place deep nulls in the desired directions. The FPA is implemented on MATLAB^®^ and executed 15 times. The number of iterations for each run is set equal to 1000. All results were obtained using *n* = 25, β = 1.5, *p* = 0.8, and γ = 0.1.

### Peak SLL minimization

The fitness function used for the minimization of peak SLL is formulated as given by (8)

8 $$Fitness = \hbox{min} \left( {\hbox{max} \left( {20\log \left| {AF\left( \theta \right){\kern 1pt} } \right|} \right)} \right)$$

#### Design example A

This example illustrates the design of 2*N* = 10 element linear array for achieving minimum SLL in the regions, *θ* = [0°,74°] and *θ* = [106°,180°]. The flower pollination algorithm with fitness function given by (8) is used for the determination of optimized element locations, *x*_*n*_. Uniform amplitude and phase excitations are assumed, i.e., *I*_*n*_ = 1 and *ψ*_*n*_ = 0.

**Fig. 3:**
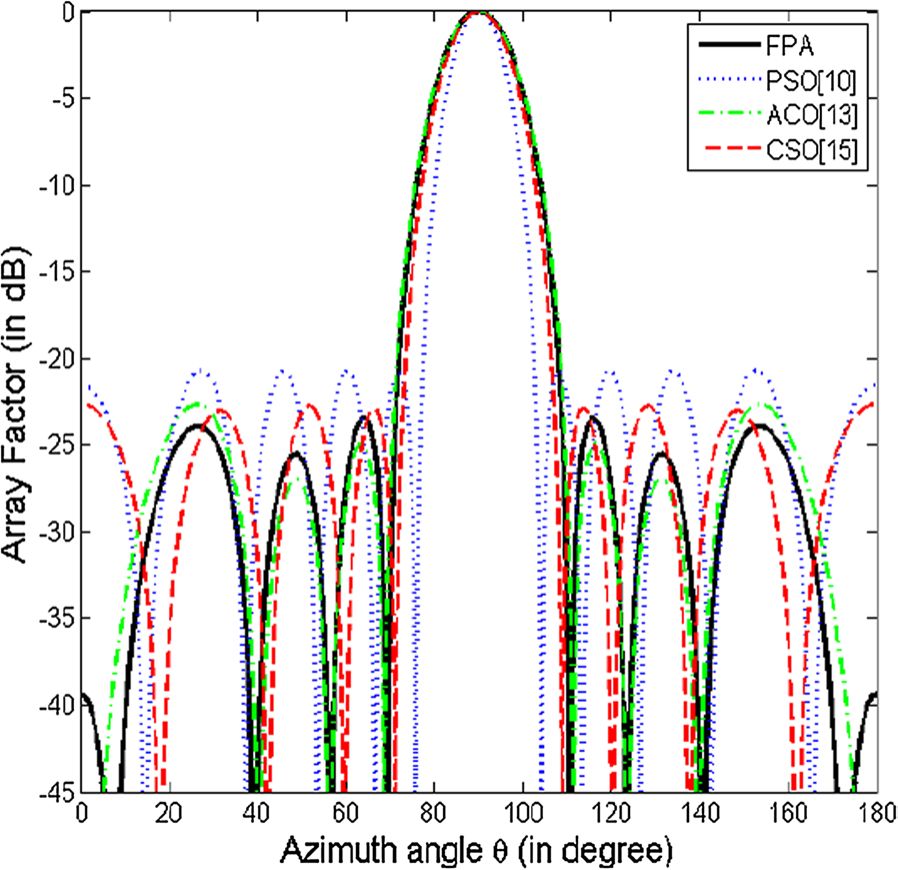
Array pattern for design example A

The optimized element positions are shown in Table 2 and the array pattern is illustrated in Fig. 3. For benchmarking purpose, the peak SLL obtained for this design example using the proposed method (FPA) and other nature-inspired optimization techniques is summarized in Table 3. In comparison to (non-optimized) conventional arrays, and arrays optimized using other opitimization algorithms such as PSO (Khodier and Christodoulou 2005), ACO (Rajo-Iglesias and Quevedo-Teruel 2007) and CSO (Pappula and Ghosh 2014), the proposed approach (FPA) shows a marked reduction in SLL.

**Table 2:** Optimized positions of the positive half of the 10 element array of design example A

Method	Optimized element positions				
CSO (Pappula and Ghosh 2014)	0.1516 λ	0.4115 λ	0.7899 λ	1.1048 λ	1.6843 λ
Proposed	0.1342 λ	0.375 λ	0.7522 λ	0.9875 λ	1.5661 λ

**Table 3:** Optimized peak SLL for 10 element linear array of design example A

Sr. no.	Approach	Peak SLL (in dB)
1	Conventional (without optimization)	−13.23
2	PSO (Khodier and Christodoulou 2005)	−20.72
3	ACO (Rajo-Iglesias and Quevedo-Teruel 2007)	−22.66
4	CSO (Pappula and Ghosh 2014)	−22.89
5	Proposed	−23.45

The proposed method (FPA) gives a peak SLL of −23.45 dB. This is 10.22 dB lower in comparison to conventional array. The peak SLL has been lowered from −20.72 to −23.45 dB (by 2.73 dB) as compared to PSO optimized array, and from −22.66 to −23.45 dB as compared to ACO optimized array, and from −22.89 to −23.45 dB as compared to CSO optimized array.

### SLL minimization along with null placement

The fitness function used for SLL minimization as well as for placement of nulls in desired directions is formulated as given in (9).

9 $$Fitness = \sum\limits_{i} {\frac{1}{{\Delta \theta_{i} }}} \int\limits_{{\theta_{li} }}^{{\theta_{ui} }} {\left| {AF\left( \theta \right){\kern 1pt} } \right|^{2} } d\theta + \sum\limits_{k} {\left| {AF\left( {\theta_{k} } \right){\kern 1pt} } \right|}^{2}$$

where, $\theta_{li}$ and $\theta_{ui}$ are the spatial regions in which SLL is suppressed and $\Delta \theta_{i}$ = $\theta_{ui}$-$\theta_{li}$. The null direction is given by $\theta_{k}$. In (9), the first term of the fitness function is for SLL suppression and the second term accounts for the placement of nulls in desired directions.

#### Design example B

**Fig. 4:**
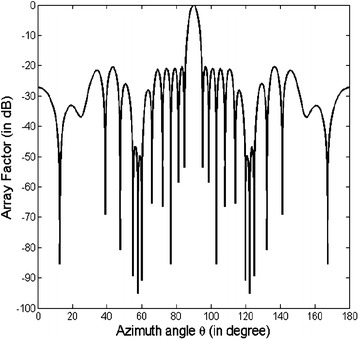
Array pattern for design example B

This design example illustrates the synthesis of 28 element linear antenna array in order to achieve SLL minimization in the regions *θ* = [0°,84°] and *θ* = [96°,180°] along with null placement at *θ* = 55°, 57.5°, 60°, 120°, 122.5° and 125°. The fitness function used by the FPA for this design example is given by (9). The array pattern is shown in Fig. 4, and the optimized positions of the antenna elements are given in Table 4. It is seen from Fig. 4 that the proposed method using FPA enables the placement of deep nulls (as deep as −95.12 dB) at desired directions.

**Table 4:** Optimized positions of the positive half of the 28 element array of design example B

Method	Optimized element positions						
CSO (Pappula and Ghosh 2014)	0.2720 λ	0.7547 λ	1.1399 λ	1.7065 λ	2.3287 λ	2.8675 λ	3.3536 λ
	3.7693 λ	4.2222 λ	4.8991 λ	5.4061 λ	5.7389 λ	6.1564 λ	6.7173 λ
Proposed	0.1515 λ	0.5415 λ	0.879 λ	1.2672 λ	1.6341 λ	2.0648 λ	2.3989 λ
	2.8480 λ	3.2977 λ	3.7082 λ	4.4512 λ	4.9587 λ	5.4789 λ	6.1333 λ

The null depths obtained by the proposed method using FPA at each of the specified directions are summarized in Table 5. The comparative analysis of minimum null depth and peak SLL obtained using the proposed method (FPA) and various other state-of-the-art optimization algorithms is shown in Table 6. It is seen that for this design example, the minimum null depth obtained by using FPA is −89.42 dB. This implies that the obtained nulls are at least as deep as −89.42 dB. There is an improvement of around 39 dB in null depth obtained using PSO (Khodier and Christodoulou 2005) and ACO (Rajo-Iglesias and Quevedo-Teruel 2007). Compared to CSO (Pappula and Ghosh 2014), the proposed FPA approach improves null depth by around 24 dB. The peak SLL obtained using the proposed method (FPA) is −20.46 dB, which is about 7.23 dB lower than conventional array and PSO optimized array (Khodier and Christodoulou 2005), about 5.46 dB lower than ACO(Rajo-Iglesias and Quevedo-Teruel 2007) optimized array, and about 7.67 dB lower than CSO optimized array (Pappula and Ghosh 2014).

**Table 5:** Null depths after optimization by FPA for design example B

Linear array type	Null depth (in dB)					
	55°	57.5°	60°	120°	122.5°	125°
28 Element array	−89.42	−95.12	−90.81	−90.81	−95.12	−89.42

**Table 6:** Comparative analysis of null depth and peak SLL obtained by various optimization algorithms for design example B

	Method			
	PSO (Khodier and Christodoulou 2005)	ACO (Rajo-Iglesias and Quevedo-Teruel 2007)	CSO (Pappula and Ghosh 2014)	Proposed
Minimum null depth (in dB)	−50	~−50	−65	−89.42
Peak SLL (in dB)	−13.23	−15	−12.79	−20.46

#### Design example C

In this design example, FPA is used to optimize the antenna element positions for SLL minimization and null placement of 32 element linear antenna array. The fitness function used by the FPA for this design example is given by (9). SLL reduction is desired in the spatial regions *θ* = [0°,85°] and *θ* = [95°,180°] whereas nulls are desired to be placed at *θ* = 81° and *θ* = 99° (very close to the first sidelobe).

**Fig. 5:**
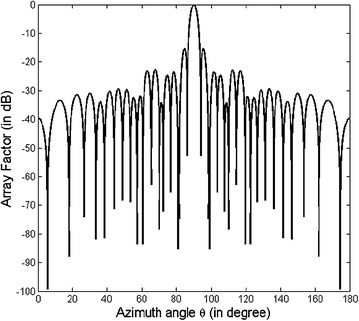
Array pattern for design example C

The array pattern is shown in Fig. 5 and the optimized positions of the antenna elements are given in Table 7. The array optimized by the proposed approach of using FPA has almost the same length as that obtained by CSO (Pappula and Ghosh 2014). It is seen from Fig. 5 that the proposed approach of using FPA enables the placement of nulls as deep as −85.27 dB at the desired directions (*θ* = 81° and *θ* = 99°); very close to the first sidelobe.

**Table 7:** Optimized positions of the positive half of the 32 element array of design example C

Method	Optimized element positions			
CSO (Pappula and Ghosh 2014)	0.2883 λ	0.6830 λ	1.1929 λ	1.5199 λ
	1.9768 λ	2.3247 λ	2.6886 λ	3.1362 λ
	3.4848 λ	3.9538 λ	4.3822 λ	4.9252 λ
	5.4817 λ	6.2091 λ	7.0412 λ	7.7500 λ
Proposed	0.25 λ	0.7496 λ	1.2498 λ	1.7467 λ
	2.2260 λ	2.6477 λ	3.0084 λ	3.4055 λ
	3.7633 λ	4.2562 λ	4.75 λ	5.2504 λ
	5.7510 λ	6.4361 λ	7.2490 λ	7.9975 λ

For this design example, PSO (Khodier and Christodoulou 2005) offers null depth of −60 dB, ACO (Rajo-Iglesias and Quevedo-Teruel 2007) gives −50 dB nulls whereas CSO (Pappula and Ghosh 2014) places deep nulls of −80 dB as seen in Table 8. However, the proposed approach (FPA) places the deepest null of −85.27 dB. The first side lobe obtained by FPA is about 3 dB higher than that obtained using CSO (Pappula and Ghosh 2014). However, the remaining sidelobes are almost similar to those obtained by using CSO (Pappula and Ghosh 2014).

**Table 8:** Comparative analysis of null depth obtained by various optimization algorithms for design example C

	Method			
	PSO (Khodier and Christodoulou 2005)	ACO (Rajo-Iglesias and Quevedo-Teruel 2007)	CSO (Pappula and Ghosh 2014)	Proposed
Null depth (in dB)	−60	−50	−80	−85.27

### Convergence of FPA

**Fig. 6:**
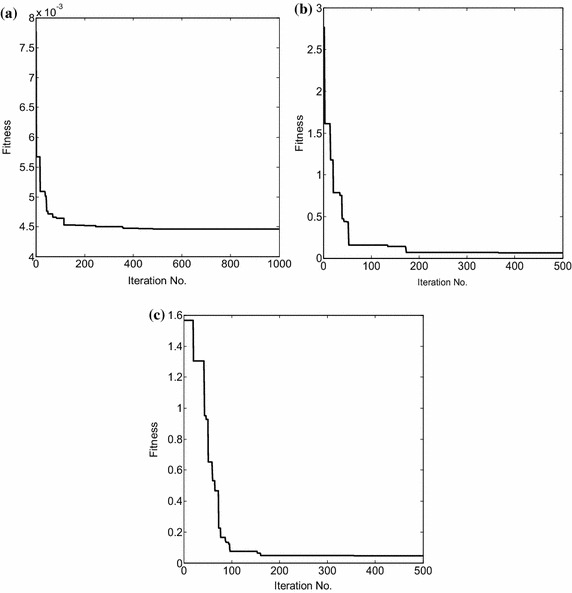
Convergence curve of FPA. **a** Design example A, **b** design example B, **c** design example C

Figure 6 shows the convergence of the fitness function versus the number of iterations for all the three design examples. The comparative relation based on the number of iterations taken by different optimization techniques to reach the optimal solution is depicted in Table 9. It is observed that although FPA is simpler to implement and also yields improved performance, it takes more number of iterations to converge on to the optimum solution as compared to PSO. In PSO, all the particles move through global search and end with local search in the last generation. The momentum effects on particle movement (e.g. when a particle is moving in the direction of a gradient) generally allow faster convergence. On the other hand, in FPA, global and local pollination techniques are carried out in each generation to create a balance between explorations and exploitations with the help of switching probability. Thus, the algorithm is more likely to escape locally optimal points, and yield a global optimum solution. FPA has to perform the process of global search, thus making it more computationally time consuming than PSO as depicted in Table 9. It is seen that FPA converges to the optimum solution much faster than ACO. ACO algorithm takes too long to converge, and also traps in local optima in order to find an optimal solution as there is no mechanism to control the randomness of ants.

**Table 9:** No. of iterations required for convergence by different optimization algorithms

Algorithm	No. of iterations required for convergence		
	Design example A	Design example B	Design example C
PSO (Khodier and Christodoulou 2005)	400	–	200
ACO (Rajo-Iglesias and Quevedo-Teruel 2007)	800	100	260
Proposed (FPA)	482	173	204

## Effect of control parameters on quality of solution

The control parameters of FPA have been tuned in order to achieve better quality of solution. This section presents the statistical results in terms of best, worst, mean and median fitness obtained by carrying out a detailed parametric study to tune the parameters of FPA.

### Effect of variation in population size (*n*)

The final fitness values corresponding to the minimum side lobe level (design example A) and to the minimum SLL and null depth (design example B and C) with variation in population size are shown in Table 10. FPA is executed 15 times for different population sizes, keeping all other parameters constant. As the population size is increased, the fitness values converge to a minimum. However, the computational time also increases with increase in population size. It is seen from Table 10 that *n* = 25 is an optimum choice, as the fitness values are minimum for this case and do not show significant change on further increase in *n*.

**Table 10:** Statistical values of the fitness function with variation in population size (*n*)

	Fitness	Population size (*n*)									
		5	10	15	20	25	30	35	40	45	50
Design example A	Best	0.0048	0.0045	0.0039	0.0036	0.0034	0.0034	0.0034	0.0034	0.0034	0.0034
	Worst	0.0067	0.0066	0.0064	0.0063	0.0063	0.0063	0.0063	0.0063	0.0063	0.0063
	Mean	0.0058	0.0056	0.0053	0.0053	*0.0051*	0.0051	0.0051	0.0051	0.0051	0.0051
	Median	0.0052	0.0051	0.0047	0.0047	*0.0045*	0.0045	0.0045	0.0045	0.0045	0.0045
Design example B	Best	0.0660	0.0533	0.0532	0.0496	0.0483	0.0483	0.0498	0.0490	0.0534	0.0539
	Worst	0.3634	0.0770	0.0804	0.0818	0.0763	0.0763	0.0763	0.0764	0.0769	0.0763
	Mean	0.1777	0.0641	0.0643	0.0651	*0.0619*	0.0620	0.0626	0.0621	0.0628	0.0648
	Median	0.1685	0.0645	0.0632	0.0684	*0.0603*	0.0632	0.0628	0.0624	0.0614	0.0661
Design example C	Best	0.0683	0.0553	0.0450	0.0438	0.0436	0.0438	0.0441	0.0438	0.0439	0.0440
	Worst	0.2249	0.5533	0.5533	0.0961	0.0463	0.0506	0.0468	0.0515	0.0524	0.0516
	Mean	0.1455	0.5533	0.5685	0.0518	*0.0442*	0.0453	0.0448	0.0453	0.0456	0.0451
	Median	0.1209	0.5533	0.5521	0.0452	*0.0438*	0.0444	0.0447	0.0449	0.0445	0.0447

Italic values represent mean and median for the optimum value of tuned parameter

### Effect of switching probability (*p*)

The final fitness values corresponding to the minimum side lobe level (design example A) and to the minimum SLL and null depth (design example B and C) with variation in switching probability are shown in Table 11. FPA is executed 15 times for the different values of switching probability, keeping all other parameters constant. FPA essentially controls the degrees of explorations and exploitations with the switching probability (*p*). Global and local pollination techniques are used to balance explorations and exploitations. A higher value of *p* is more likely to explore the search space globally and escape from local minima points. It is seen from Table 11 that *p* = 0.8 is a good choice since it offers minimum value of fitness function. However, if *p* is increased the quality of solution degrades. This is because it leads to too much exploration at the cost of too little exploitation, which in turn compromises the overall search performance.

**Table 11:** Statistical values of the fitness function with variation in switching probability (*p*)

	Fitness	Switching probability (*p*)								
		0.1	0.2	0.3	0.4	0.5	0.6	0.7	0.8	0.9
Design example A	Best	0.0066	0.0058	0.0051	0.0048	0.0045	0.0039	0.0036	0.0034	0.0039
	Worst	0.0089	0.0074	0.0070	0.0067	0.0066	0.0064	0.0063	0.0063	0.0064
	Mean	0.0078	0.0069	0.0062	0.0058	0.0056	0.0053	0.0053	*0.0051*	0.0053
	Median	0.0072	0.0063	0.0056	0.0052	0.0051	0.0047	0.0047	*0.0045*	0.0047
Design example B	Best	0.0668	0.0533	0.0561	0.0516	0.0548	0.0486	0.0489	0.0483	0.0570
	Worst	0.0834	0.0789	0.0770	0.0765	0.0764	0.0764	0.0763	0.0763	0.0944
	Mean	0.0743	0.0669	0.0627	0.0624	0.0624	0.0624	0.0624	*0.0619*	0.0786
	Median	0.0747	0.0659	0.0636	0.0634	0.0639	0.0671	0.0603	*0.0603*	0.0794
Design example C	Best	0.0439	0.0437	0.0436	0.0436	0.0436	0.0436	0.0437	0.0436	0.0446
	Worst	0.0495	0.0473	0.0495	0.0463	0.0487	0.0481	0.0513	0.0463	0.1423
	Mean	0.0458	0.0444	0.0443	0.0443	0.0443	0.0443	0.0442	*0.0442*	0.0682
	Median	0.0460	0.0441	0.0441	0.0442	0.0438	0.0438	0.0438	*0.0438*	0.0504

Italic values represent mean and median for the optimum value of tuned parameter

### Effect of *β*

*β* is the index used in Levy distribution for generating Levy-flights for global pollination. The final fitness values corresponding to the minimum side lobe level (design example A) and to the minimum SLL and null depth (design example B and C) with variation in *β* are shown in Table 12. FPA is executed 15 times for different values of *β* while keeping all other parameters constant. It is seen that *β* = 1.5 is a good choice as it gives the lowest value of fitness function. For small *β*, random walks tend to get crowded around a central location, and occasionally jump quite a big step to a new location. As *β* increases, the probability of performing a long jump decreases. For *β* = 1, the Levy distribution reduces to the Cauchy distribution, and for *β* = 2, a Gaussian distribution is obtained. As *β* varies from 1 to 2, the Levy distribution varies from Gaussian to Cauchy, and the tail probabilities vary from light to heavy. This makes *β* = 1.5 a good choice for an intermediate Levy distribution and Levy flight.

**Table 12:** Statistical values of the fitness function with variation in *β*

	Fitness	*β*				
		1	1.25	1.5	1.75	1.9
Design example A	Best	0.0039	0.0036	0.0034	0.0036	0.0038
	Worst	0.0064	0.0063	0.0063	0.0064	0.0063
	Mean	0.0053	0.0053	*0.0051*	0.0053	0.0056
	Median	0.0047	0.0047	*0.0045*	0.0047	0.0049
Design example B	Best	0.0650	0.0542	0.0483	0.0518	0.0488
	Worst	0.0883	0.0820	0.0763	0.0765	0.0765
	Mean	0.0742	0.0678	*0.0619*	0.0649	0.0666
	Median	0.0713	0.0678	*0.0603*	0.0677	0.0692
Design example C	Best	0.0436	0.0436	0.0436	0.0438	0.0438
	Worst	0.3511	0.0506	0.0463	0.1422	0.3509
	Mean	0.0644	0.0452	*0.0442*	0.0639	0.0713
	Median	0.0439	0.0442	*0.0438*	0.0499	0.0470

Italic values represent mean and median for the optimum value of tuned parameter

## Conclusion

This paper introduced the flower pollination algorithm for the optimization of linear antenna arrays. FPA was applied to obtain optimized antenna positions in order to achieve desired array pattern with minimum SLL along with null placement in specified directions.

Design examples were presented for the following conditions: peak SLL suppression (design example A), SLL minimization along with placement of multiple nulls close to each other in the spatial region (design example B), and SLL minimization along with placement of nulls close to the first side lobe (design example C). The obtained results have been compared with conventional array (non-optimized), and with arrays optimized using other nature-inspired evolutionary algorithms such as PSO, ACO and CSO. The results indicate that FPA yields improved performance in peak SLL suppression as well as in terms of placement of strong nulls in desired directions along with SLL minimization. In the field of antenna array optimization, FPA’s performance demonstrates its suitability for the antenna and electromagnetics community.
